# Genome–Wide Identification of the GRAS Family Genes in *Melilotus albus* and Expression Analysis under Various Tissues and Abiotic Stresses

**DOI:** 10.3390/ijms23137403

**Published:** 2022-07-03

**Authors:** Shengsheng Wang, Zhen Duan, Qi Yan, Fan Wu, Pei Zhou, Jiyu Zhang

**Affiliations:** State Key Laboratory of Grassland Agro–Ecosystems, Key Laboratory of Grassland Livestock Industry Innovation, Ministry of Agriculture and Rural Affairs, College of Pastoral Agriculture Science and Technology, Lanzhou University, Lanzhou 730020, China; shshwang21@lzu.edu.cn (S.W.); duanzh12@lzu.edu.cn (Z.D.); yanq16@lzu.edu.cn (Q.Y.); wuf15@lzu.edu.cn (F.W.); zhoup2017@lzu.edu.cn (P.Z.)

**Keywords:** *Melilotus albus*, GRAS transcription factor, bioinformatics, stress response, gene expression

## Abstract

The GRAS gene family is a plant–specific family of transcription factors, which play an important role in many metabolic pathways, such as plant growth and development and stress response. However, there is no report on the comprehensive study of the GRAS gene family of *Melilotus albus*. Here, we identified 55 *MaGRAS* genes, which were classified into 8 subfamilies by phylogenetic analysis, and unevenly distributed on 8 chromosomes. The structural analysis indicated that 87% of *MaGRAS* genes have no intron, which is highly conservative in different species. MaGRAS proteins of the same subfamily have similar protein motifs, which are the source of functional differences of different genomes. Transcriptome and qRT–PCR data were combined to determine the expression of 12 *MaGRAS* genes in 6 tissues, including flower, seed, leaf, stem, root and nodule, which indicated the possible roles in plant growth and development. Five and seven *MaGRAS* genes were upregulated under ABA, drought, and salt stress treatments in the roots and shoots, respectively, indicating that they play vital roles in the response to ABA and abiotic stresses in *M. albus*. Furthermore, in yeast heterologous expression, *MaGRAS12*, *MaGRAS34* and *MaGRAS33* can enhance the drought or salt tolerance of yeast cells. Taken together, these results provide basic information for understanding the underlying molecular mechanisms of GRAS proteins and valuable information for further studies on the growth, development and stress responses of GRAS proteins in *M. albus*.

## 1. Introduction

GRAS gene family is a plant–specific transcription factor family, the name GRAS is derived from the three functional members, gibberellic acid insensitive (GAI) [1], repressor of GAI (RGA) [2] and scarecrow (SCR) [3], which are widely distributed in plants. Members of the GRAS protein family are quite different in length and nucleotide sequence, and GRAS proteins typically consist of 400–770 amino acids [4]. Their C–terminal sequences are highly conserved, mainly including five motifs: LHR I (Leucine Heptapeptide Repeat I), VHIID, LHR II (Leucine Heptad Region II), PFYRE and SAW, among which VHIID conserved sequence is the core sequence of GRAS family proteins; these motifs are crucial to the interaction between GRAS and other proteins [5]. In contrast, the N–terminal part of the GRAS proteins is highly variable and will fold specifically when it meets a suitable ligand [6]. Studies have shown that the N–terminal sequence plays an important role in the specific function of GRAS proteins [7]. The phylogenetic tree can divide GRAS family into several subfamilies, but the number of subfamilies in different species is also different. According to the classification method of the *Arabidopsis thaliana* GRAS gene family, it is divided into eight subfamilies, including LISCL, PAT1, SCL3, DELLA, SCR, SHR, LAS and HAM [8]. These subfamilies are all named after their star members or unified functions. GRAS proteins from various subfamilies have distinct functions.

At present, genome–wide members of the GRAS gene family have been identified in a number of plant species, and 34, 60, 153 and 68 GRAS gene members were found from *A. thaliana* [9], rice (*Oryza sativa*) [9], wheat (*Triticum aestivum*) [10] and *Medicago truncatula* [11], respectively. GRAS proteins play various roles in plant growth and development, and participate in phytohormone signal transduction and the regulation of tissue development. [4]. SCR and SHR subfamily members are closely related to plant root growth [12]. SCR and SHR form a SCR/SHR complex, which plays an essential role in the extension direction of the plant root network [13]. SCL3 participates in Gibberellic acid/DELLA regulation pathway and cooperates with SHR/SCR to mediate cell elongation during root development [14]. The SHR/SCR/SCL3 module of GRAS transcription factor in tomato (*Solanum lycopersicum*) participates in the regulation of gibberellin signaling during mycorrhization process [15]. DELLA proteins are the key negative regulator of Gibberellic acid signaling pathway, and it mediates the synergistic regulation of gibberellin and light signal by interacting with PIF protein [16]. In addition, it is found that DELLA proteins participate in signal transmission and regulation of various hormones as a core role in the plant hormone regulation network, for example, in *A. thaliana*, the DELLA proteins control plant immune response by regulating the balance of jasmonic acid and salicylic acid signaling [17]. RGA–LIKE 1 (RGL1), RGL2, RGL3, GAI and RGA have conserved sequences, which are sensed by gibberellin signals and can participate in gibberellin signal regulation [18]. PAT1, which is regulated by DELLA protein, participates in gibberellin signal mediation and interacts with SCL3 protein to regulate photosensitive pigment signal transduction [19]. *AtPAT1* in *A. thaliana* plays a positive role in regulating the signal transduction of phytochrome A [20]. Studies have suggested that GRAS proteins are related to nodule formation of leguminous plants [21]. *NSP1* (nodulation signaling pathway 1) and *NSP2* (nodulation signaling pathway 2) are two GRAS proteins identified from *M. truncatula*, belonging to SHR and HAM subfamilies, respectively [22]. They can directly bind to promoters of nodulation–related genes and play an important role in nodule formation, which shows the importance of GRAS proteins for the effective nodulation of legumes [21].

In addition, GRAS proteins have been involved in the plant response to biotic or abiotic stress. LISCL subfamily plays an important role in plants responding to stress. SCL14 is a LISCL protein in *A. thaliana*, and forms the TGA/SCL14 complex with TGA transcription factor, which is essential for activating stress–induced promoters when plants are attacked by exogenous substances [23]. *VaPAT1* in *Vitis amurensis* plays a role as a positive transcription regulator in abscisic acid (ABA) and various abiotic stresses [24]. In poplar (*Populus euphratica* Oliv), *PeSCL7* is induced by drought and high salt stresses, which may be useful for engineering drought–resistant and salt–tolerant trees [25]. The expression of *OsGRAS23* gene in rice was induced by drought, NaCl and jasmonic acid, and its overexpressed plants enhanced drought tolerance by reducing H_2_O_2_ accumulation in cells [26]. Overexpressing *BnLAS* gene from *Brassica napus* in *A. thaliana* plants significantly enhanced drought resistance by increasing wax secretion in leaves and reducing stomatal opening [27]. In tomato, the expression levels of *SlGRAS7* and *SlGRAS40* were significantly upregulated under salt and D–mannitol treatment, and transgenic tomato plants overexpressing *SlGRAS7* and *SlGRAS40* were more tolerant to drought and salt stress than wild–type plants [28,29]. Moreover, *GRAS* genes were also found to respond to multifarious hormones [30]. The *GRAS* genes *PgGRAS44–04*, *PgGRAS48–01* and *PgGRAS50–01* in *Panax ginseng* are expressed under gibberellic acid treatment [31]. The downregulation of *GRAS2* in tomato decreased the activity of gibberellic acid biosynthesis and signal transduction pathway, thus reducing the fruit weight of tomato [32].

*Melilotus albus* is a leguminous crop, which is an important forage and green manure around world [33]. It has strong stress resistance, especially its salt resistance and drought resistance [34]. It is one of the excellent forage legume for soil improvement [35]. In recent years, more attention has been paid to the research of GRAS gene family in plant species growth, signal transduction and abiotic stresses response. However, the whole genome analysis and function of GRAS family have not been reported in *M. albus*. In this study, using the latest available genome assembly and annotation database of *M. albus* [36], we comprehensively analyzed 55 *GRAS* genes by investigating physical and chemical properties, phylogenetic relations, gene duplication events, gene structures, motif compositions, chromosomal locations and cis–elements in promoter regions. Moreover, we explored the expression patterns of the *MaGRAS* genes in different tissues, ABA treatment, salt and drought stresses. In particular, six representative *MaGRAS* genes were selected and carried out the qRT–PCR analyses, and three of them were further selected to analyze their drought and salt tolerance functions by heterologous expression in yeast. Collectively, our results provide a theoretical basis for the role of *GRAS* genes in the abiotic stresses response of leguminous plants. 

## 2. Results

### 2.1. Identification and Analysis of GRAS Genes in Melilotus albus

To identify *GRAS* genes in the *M. albus* genome, we conducted blastp searches on the *M. albus* genome using GRAS sequences documented in *A. thaliana* and *M. truncatula* as query sequences. Using this method, 172 putative *GRAS* genes were identified and submitted to CDD, Pfam and SMART to confirm the GRAS domain. A total of 55 genes were identified as predicted by *GRAS* genes. The 55 *MaGRAS* genes were named according to their physical positions on chromosomes (from top to bottom). Gene names, gene IDs, chromosomal locations, number of amino acids, molecular weights (Mws), isoelectric points (pIs), grand average of hydropathicity (GRAVY), CDS lengths and subgroups were listed in Supplementary Table S1. The length of amino acid sequences encoded by MaGRAS varied from 155 (MaGRAS50) to 819 (MaGRAS15) amino acids, the relative Mws ranged from 17.1 (MaGRAS50) to 90.5 (MaGRAS15) kDa, the pIs values of MaGRAS proteins varied from 4.81 (MaGRAS5) to 7.60 (MaGRAS31), the GRAVY values ranged from −0.693 (MaGRAS29) to −0.116 (MaGRAS51), the CDSs were distributed from 468 to 2460 bp (Supplementary Table S1). The predicted subcellular localizations of the MaGRAS proteins showed that 36 MaGRAS members might be in the nucleus, 9 MaGRAS proteins were anchored in the chloroplast and cytoplasm, respectively, and 1 MaGRAS protein was anchored in the mitochondria (Supplementary Table S1). 

### 2.2. Phylogenetic Categories Analysis of MaGRAS Genes

**Figure 1:**
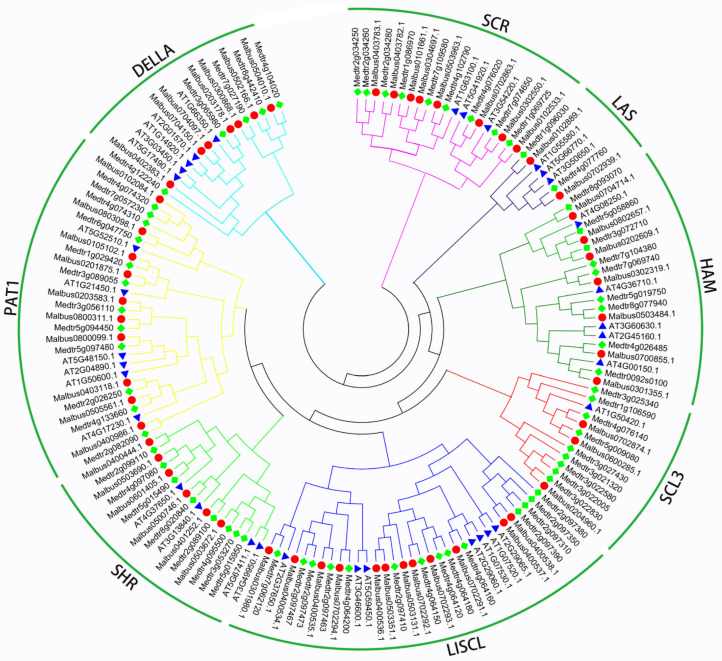
Phylogenetic tree constructed using GRAS proteins from *M. albus* (red circle), *M. truncatula* (green square) and *A. thaliana* (blue triangle). The phylogenetic tree was constructed using MEGA 7.0 and using the neighbor–joining method.

To explore the evolutionary relationship of the MaGRAS proteins and other known GRAS proteins, a phylogenetic tree was resolved using 157 GRAS family members from *M. albus* (55 genes), *M. truncatula* (68 genes) and *A. thaliana* (34 genes) (Figure 1) using the neighbor–joining (NJ) method in MEGA 7.0. All GRAS members were divided into eight subfamilies based on the previous classification of GRAS families in *A. thaliana*. The 157 *GRAS* genes from 3 species were unevenly clustered into 8 groups, including SHR, LAS, DELLA, HAM, LISCL, PAT1, SCL3 and SCR subbranches. Compared with the number of *GRAS* genes in *A. thaliana*, the GRAS gene family of *M. truncatula* and *M. albus* expanded significantly. LISCL subfamily has the largest number of *GRAS* genes, followed by PAT1 subfamily. There were seven (*A. thaliana*) *GRAS*s, 12 (*M. albus*) *GRAS*s and 14 (*M. truncatula*) *GRAS*s in the LISCL subfamily. Six (*A. thaliana*) *GRAS*s, 10 (*M. albus*) *GRAS*s and 12 (*M. truncatula*) *GRAS*s in the PAT1 subfamily. Especially, both the *A. thaliana* and *M. truncatula* contained five *GRAS*s while *M. albus* contained seven *GRAS*s in the DELLA. In addition, we identified six, eight, three, seven and two *MaGRAS* genes in the SHR, SCR, SCL3, HAM and LAS subfamilies, respectively.

### 2.3. Chromosomal Locations and Gene Duplication Analysis of MaGRAS Genes

**Figure 2:**
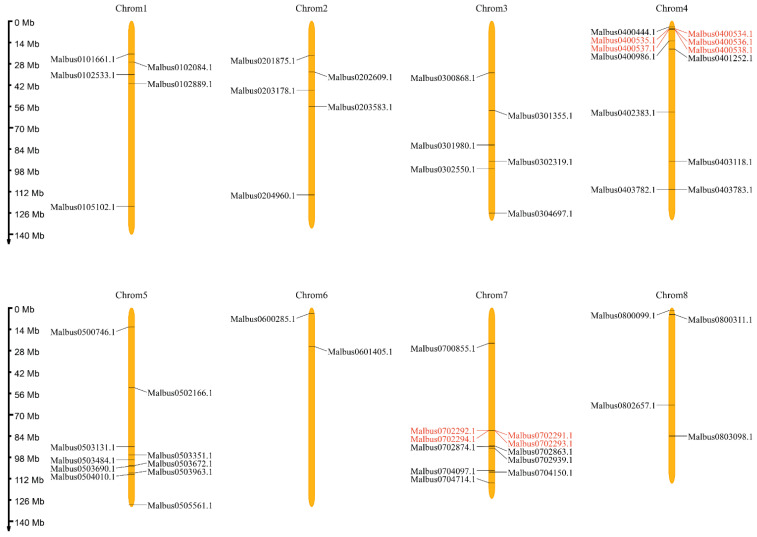
Chromosomal distribution and position of 55 *MaGRAS* genes identified in the *M. albus* genome. Eight chromosomes are indicated in orange columns, and black lines indicate the position of each *MaGRAS* genes. Red lines represent the tandemly duplicated genes.

The physical locations of 55 *MaGRAS* genes were distributed on 8 chromosomes of *M. albus* (Figure 2), showing a heterogeneous distribution. Each chromosome in *M. albus* contained between two and twelve *MaGRAS* genes. Chromosome 4 had the highest number of *MaGRAS* genes (12 genes), and most of them are distributed at the front of the chromosome. Chromosome 6 had the least number of *MaGRAS* genes (two genes), which are distributed at the front of the chromosome. Chromosomes 3 and 8 contained six and four genes, respectively, evenly distributed on the chromosome. There are five *MaGRAS* genes on chromosomes 1 and 2, and their distribution trends are consistent. Chromosomes 5 and 7 contained 10 and 11 genes, respectively, mainly located at the rear end of the chromosome.

**Figure 3:**
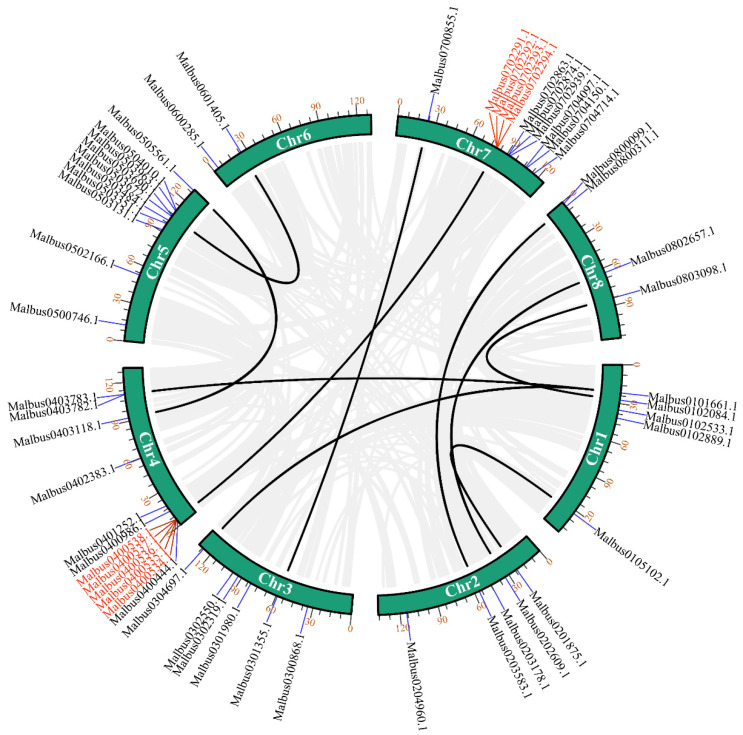
Distribution and synteny analysis of *MaGRAS* genes. In the figure, the 8 *M. albus* chromosomes are shown in green–colored partial circles, gene IDs are indicated at the top of each bar. Background gray lines indicate all *M. albus* genome synteny blocks, black lines indicate the duplicated *MaGRAS* genes, red lines represent the tandemly duplicated *MaGRAS* genes.

We used TBtools software for collinearity analysis to detect the gene duplication of *MaGRAS* genes in *M. albus*. *MaGRAS* genes had obvious tandem repeat phenomenon (Figure 3), a total of 10 pairs of duplicated segments in *MaGRAS* genes and 3 groups of tandemly duplicated *MaGRAS* genes (*Malbus0400534.1*/*Malbus0400535.1*, *Malbus0400538.1*/*Malbus0400536.1*/*Malbus0400537.1*, *Malbus0702293.1*/*Malbus0702291.1*/*Malbus0702294.1*/*Malbus0702292.1*), 2 of which are located on chromosome 4 and 1 on chromosome 7.

### 2.4. Structure and Conserved Motifs of MaGRAS Genes

**Figure 4:**
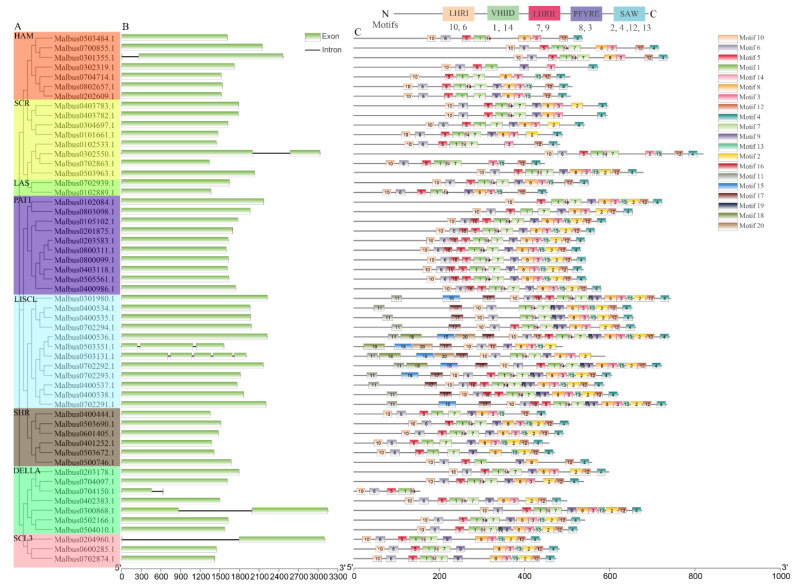
Exon–intron structure and distribution of conserved motifs of *MaGRAS* genes in *M. albus*: (**A**), MaGRAS proteins are categorized into 8 subfamilies, which are classified and labeled with different colors, including LISCL, SHR, PAT1, LAS, HAM, DELLA, SCR, and SCL3; (**B**), exon–intron structures of *MaGRAS* genes. Intron indicated by black line and CDS exon indicated by green boxes; (**C**), schematic diagram of the conserved motifs in the MaGRAS proteins. Each motif is represented by a number in the colored box. The black lines represent the non–conserved sequences. A total of 5 conserved domains and corresponding motifs in the GRAS proteins sequences are shown at the top. A scale of gene and protein length is shown at the bottom.

A single rootless phylogenetic tree was generated from the complete protein sequences of all *GRAS* genes in *M. albus*, and *MaGRAS* genes were divided into eight subgroups. (Figure 4A). Gene structural diversity is an important basis for the evolution of gene families [37]. To further understand the structural evolution of the *MaGRAS* genes, the genome sequence and CDS sequence information of each *MaGRAS* gene were obtained according to the *M. albus* genome information, and the exon–intron structure diagram was drawn (Figure 4B). The results showed that the number of exons and introns of each member of MaGRAS family was kept relatively constant, and most of the *MaGRAS* genes (87%) contained only exons and no introns. It is indicated that no introns are the main structural forms of *MaGRAS* genes. *MaGRAS* genes in the same subgroup of phylogenetic tree showed a similar exon–intron structure; *MaGRAS* genes of groups LAS, PAT1, SHR, GRAS8 and DELLA were highly conserved without introns, groups HAM (*Malbus0301355.1*), SCR (*Malbus0302550.1*) and SCL3 (*Malbus0204960.1*) each have a gene with only one intron, group DELLA (*Malbus0704150.1*, *Malbus0300868.1*) has two genes with only one intron, group LISCL has two genes with two (*Malbus0503351.1*) and four (*Malbus0503131.1*) introns.

In order to further reveal the diversity of *MaGRAS* genes, MEME program predicted the putative motifs and identified 20 different motifs (Figure 4C). In addition, the detailed information and Seq logo of 20 MEME motifs are shown in Supplementary Table S2 and Figure S1. The results showed that most closely related members in the phylogenetic tree had similar motifs. The MEME motifs were further identified and classified into five GRAS–specific C–terminal domains, including LRHI, VHIID, LRHII, PFYRE and SAW [38]. As a result, motifs 6 and 10 classified into the LRHI, motifs 1 and 14 belonged to the VHIID, motifs 7 and 9 related with the LRHII, motifs 8 and 3 were included by the PFYRE and motifs 2, 4, 12 and 13 in the SAW were shared across almost all *MaGRAS* genes. In addition, motifs 5 and 16 were located between the LRHI and VHII D, motif 5 exists in most MaGRAS subfamilies and motif 16 exists specifically in PAT1 (eight genes) and LISCL subfamily (four genes), indicating their functional importance. It was worth noting that the MEME motifs among the five C–terminal domains was not fixed in MaGRAS proteins, and some C–terminal conserved domains were corresponding to two or three motifs. Interestingly, other motifs located outside the C–terminal conserved domains showed subgroup–specific patterns. For example, the motifs 11, 15, 17, 18, and 20 were only found in the LISCL subfamily, while motif 19 was nested within LHRII and was LISCL and DELLA subfamilies specific.

In addition, based on the interaction of GRAS proteins in *M. truncatula*, we predicted the interaction of different GRAS protein subfamilies in *M. albus* (Supplementary Figure S2). We found that *MaGRAS14* gene of HAM subfamily was predicted to interact with proteins of multiple subfamilies, including SHR subfamily (two genes), DELLA subfamily (two genes), PAT1 subfamily (three genes), SCL3 subfamily (three genes) and SCR subfamily (four genes). Meanwhile, we predicted that *MaGRAS29*, the orthologous gene of nodulation–related transcription factor *MtNSP1*, can interact with *MaGRAS51*. The interactions between the putative orthologous of *MaGRAS3*, *MaGRAS46*, *MaGRAS17* and *MaGRAS35* have been validated in other species [39,40].

### 2.5. Identification of Cis–Elements in the MaGRAS Gene Promoters

**Figure 5:**
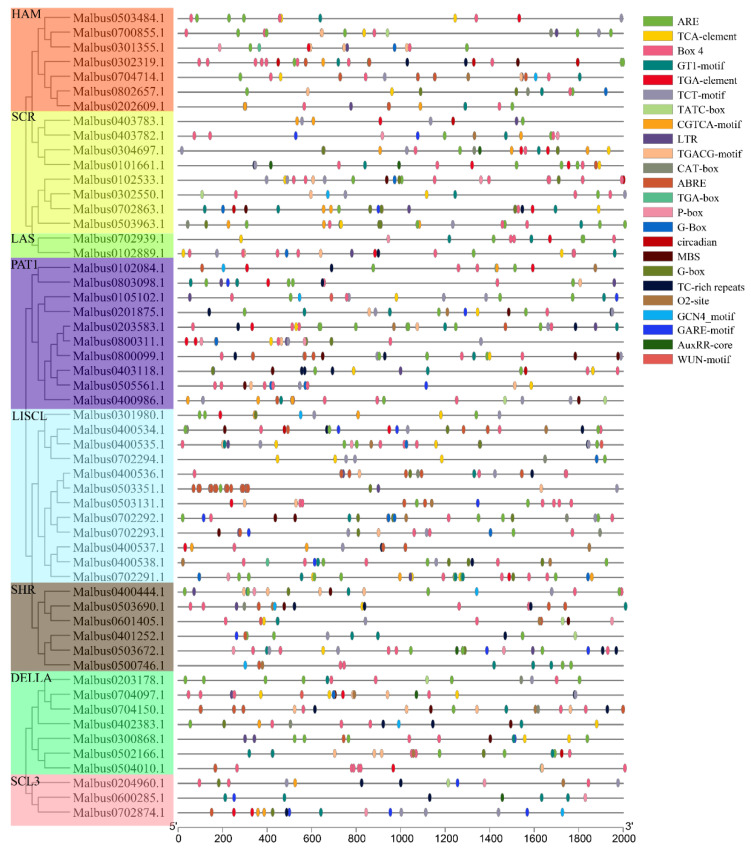
The cis–acting elements of promoter sequences (−2000 bp) of 55 *MaGRAS* genes are analyzed by PlantCARE in *M. albus*.

In order to explore the mechanism of *MaGRAS* genes in the process of stress response and development, the 2000 bp upstream sequence of *MaGRAS* genes was analyzed by PlantCARE online tools. A total of 23 elements were recorded in this study (Figure 5), which were predicted to be involved in developmental regulation, abiotic stresses and phytohormone response. Among them, there are 10 phytohormone response elements (36.5%), namely TGA–element/AuxRR–core/TGA–box (auxin–responsive elements), ABRE (abscisic acid responsive elements), GARE–motif/P–box/TATC–box (gibberellin–responsive elements), TCA–element (salicylic acid responsive elements) CGTCA–motif/TGACG–motif (methyl jasmonate responsive elements), nine defense and stress responsive elements (60.3%) of Box 4/GT1–motif/TCT–motif/G–box (light responsive elements), ARE (anaerobic induction elements), TC–rich repeats (defense and stress elements), LTR (low–temperature responsive elements), MBS (drought–inducibility elements), WUN–motif (wound–responsive elements) and 4 growth and development regulating elements (6.2%) of CAT–box (meristem expression elements), circadian (circadian control elements), GCN4_motif (endosperm expression elements) and O_2_–site (zein metabolism regulation elements). All the 55 *MaGRAS* genes contained both phytohormone response elements and stress responsive elements. *Malbus0503351.1* contained the largest number of components, 45 in total, and *Malbus0600285.1* contained the least number of components, which was only 8. The number of defense and stress responsive elements is the highest, and 397 light responsive elements were identified, which was more than the number of other cis–acting elements, followed by anaerobic induction and methyl jasmonate responsive elements.

### 2.6. Expression Pattern Analysis of MaGRAS Genes in M. albus tissues

**Figure 6:**
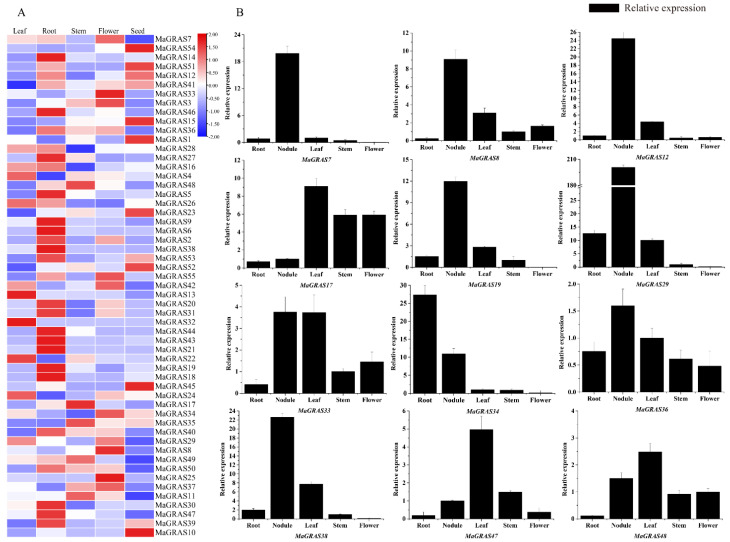
Expression analysis of the *MaGRAS* genes in different tissues: (**A**) a Heat map of all *MaGRAS* genes in different tissues based on transcriptome datasets, the expression values (FPKM) were normalized; (**B**) expression analysis of the *MaGRAS* genes in different tissues using qRT–PCR, the values shown are the means ± standard deviation of three replicates.

*GRAS* genes have important roles in various biological pathways, tissue–specific expression is related with the specific function of *MaGRAS* genes in different tissues. The expression profiles of *MaGRAS* genes in five tissues (leaf, root, stem, flower and seed tissues) were investigated (Figure 6A). A total of 41 (74.5%) *MaGRAS* genes were expressed at least in 1 tissue (FPKM ≥ 1), and 27 (49.1%) genes were expressed in all tissues (FPKM ≥ 1) (Supplementary Table S3). Among the 55 genes, 11, 15, 16, 17 and 25 *MaGRAS* genes were preferentially expressed in leaves, seeds, flowers, stems and roots, respectively (Supplementary Figure S3), and *MaGRAS8*, *MaGRAS12*, *MaGRAS23*, *MaGRAS33*, *MaGRAS49* and *MaGRAS52* were highly expressed in all tissues (FPKM ≥ 10), indicating their important role in the development of *M. albus*. 

In addition, we performed qRT–PCR analysis of 12 *MaGRAS* genes in 5 tissues (leaf, root, stem, flower and nodule tissues) during the flowering stage of *M. albus* (Figure 6B). The qRT–PCR results had similar trends to transcriptome data. *MaGRAS7, MaGRAS8*, *MaGRAS12*, *MaGRAS19*, *MaGRAS29*, *MaGRAS36* and *MaGRAS38* were significantly more highly expressed in nodules than in other tissues. The expression of *MaGRAS17* in leaves, stems and flowers were significantly higher than those in roots and nodules. *MaGRAS33* was highly expressed in leaves and nodules, but the lowest in leaves. *MaGRAS34* had the highest expression in roots, followed by in nodules. *MaGRAS47* and *MaGRAS48* were more highly expressed in leaves.

### 2.7. Expression Analysis of MaGRAS Genes Responding to ABA and Abiotic Stresses

**Figure 7:**
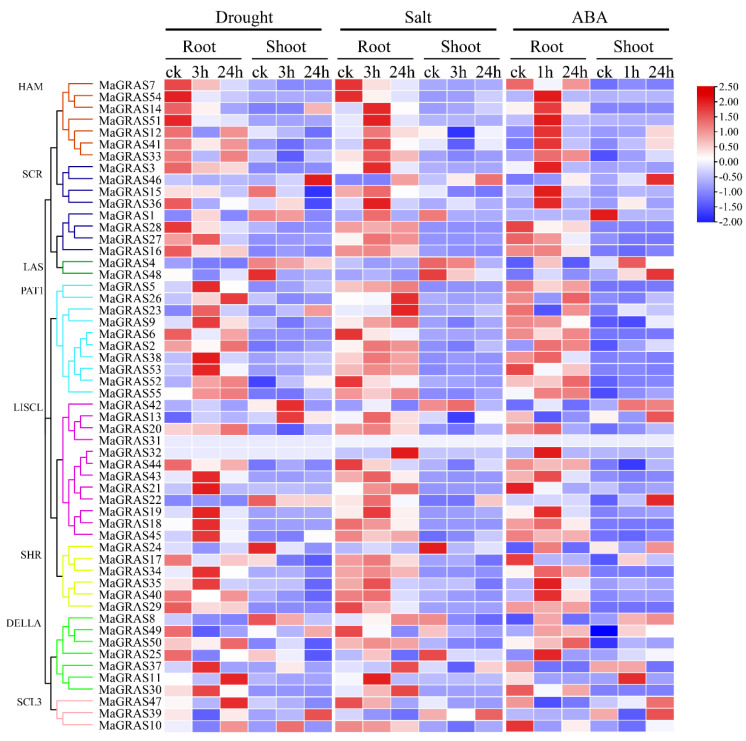
Expression of 55 *MaGRAS* genes in response to drought, salt and ABA treatments. Data were retrieved from transcriptome datasets, and the clustering was performed using TBtools. The heat map shows the relative transcript level of *MaGRAS* genes under drought, salt and ABA stresses. The expression values (FPKM) were normalized.

GRAS genes are involved in the response of plants to abiotic stresses, such as drought and salt [30]. In this study, the transcriptome data sets of *M. albus* treated with ABA, salt and drought stresses were used to explore the functions of *MaGRAS* genes under abiotic stresses. As a result, we determined that 48 (87.3%) and 35 (63.6%) *MaGRAS* genes were expressed in roots and shoots under at least 1 stress condition (FPKM ≥ 1), respectively. In shoots and roots under ABA and abiotic stress conditions, 29 (52.7%) and 22 (40.0%) *MaGRAS* genes were expressed (FPKM ≥ 1), respectively, and 13 (23.6%) and 2 (3.6%) *MaGRAS* genes were highly expressed (FPKM ≥ 10), respectively (Supplementary Table S4). The expression of 32 (19 in roots; 13 in shoots), 34 (25 in roots; 12 in shoots), and 49 (25 in roots; 24 in shoots) *MaGRAS* genes was upregulated under ABA, salt and drought stresses, respectively. Notably, five and seven *MaGRAS* genes were upregulated under ABA and abiotic stress conditions in the roots and shoots, respectively (Figure 7).

Compared with the control, among the 55 expressed *MaGRAS* genes, 27, 23 and 14 differentially expressed *MaGRAS* genes were identified in the roots of *M. albus* under drought, salt and ABA stresses, respectively. A total of 29, 26 and 34 differentially expressed *MaGRAS* genes were identified in the shoots of *M. albu*s under drought, salt and ABA stresses, respectively (Supplementary Figure S4). Meanwhile, we identified five and ten *MaGRAS* genes that were differentially expressed under three stress conditions in the roots and shoots, respectively. Fourteen and five *MaGRAS* genes were differentially expressed in shoots and roots under drought and salt stress, respectively. Two and eight *MaGRAS* genes were differentially expressed in shoots and roots under ABA and salt stress, respectively, and four and ten *MaGRAS* genes were differentially expressed in shoots and roots under ABA and drought stress, respectively, which indicated that many *MaGRAS* genes were involved in the response to abiotic stresses with independent on ABA. We determined that 46 (83.6%) *MaGRAS* genes were differentially expressed in roots and shoots under drought, salt and ABA stresses, and these genes were evenly distributed in 8 subfamilies. The other genes showed subfamily–specific characteristics, and the expression of *MaGRAS* genes clustered in PAT1 and LISCL subfamilies were highly upregulated under ABA, drought and salt stresses. The expression of most genes clustered in HAM and SCR subfamilies was highly upregulated under ABA and salt stress, while the expression of most genes clustered in SHR and DELLA subfamilies was only highly upregulated under ABA stress.

**Figure 8:**
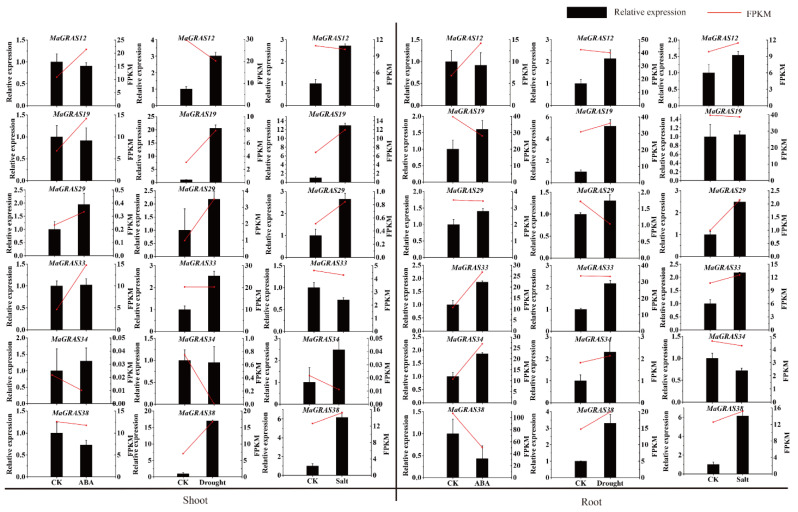
Gene expression analysis of six *MaGRAS* genes in shoots and roots under drought, salt and ABA treatments using qRT–PCR. CK represents control. Red lines indicated the expression values (FPKM) from RNA–seq data and the displayed values show the means ± standard deviation of three replicates.

In addition, in order to further verify the RNA–Seq data, six *MaGRAS* genes responding to ABA, drought and salt stresses were selected for qRT–PCR validation. The expression trends of most *MaGRAS* genes tested are consistent with the results of RNA–Seq analysis (Figure 8). Most of the tested genes were up–regulated under drought, salt and ABA stresses, indicating that they were positive regulatory genes, for example, *MaGRAS1*9 and *MaGRAS29* were upregulated in shoots and roots under drought and salt stresses, but *MaGRAS38* was downregulated in shoots and roots under ABA stress, which was similar to the RNA–Seq analysis results. These results indicated that *MaGRAS* genes play an important regulatory role in dealing with drought, salt stresses and ABA treatment.

### 2.8. MaGRAS Genes Improved the Tolerance of Yeast to Abiotic Stresses

**Figure 9:**
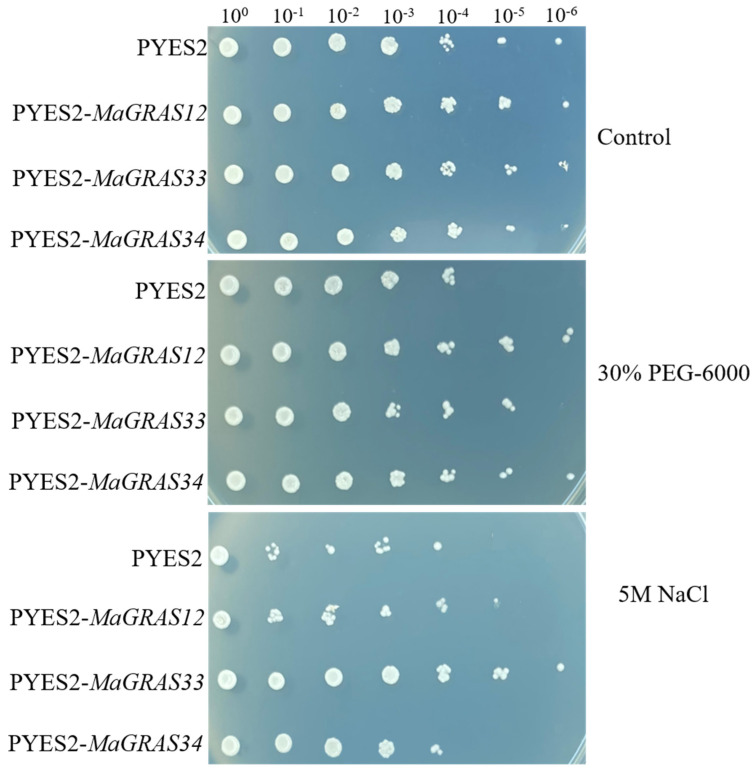
Drought and salt stresses tolerance analysis of *MaGRAS12*, *MaGRAS33* and *MaGRAS34* in a yeast expression system, using yeast with empty pYES2 vector as control.

The results of qRT–PCR showed that the expression levels of *MaGRAS12*, *MaGRAS33* and *MaGRAS34* were upregulated under drought and salt stress. In order to further verify the functions, the drought and salt tolerance functions of these genes were discussed by yeast heterologous expression analysis (Figure 9). The results showed that transgenic yeast cells of three *MaGRAS* genes and yeast cells transformed with empty vectors grew well and no difference under the control treatment. Meanwhile, we observed that the yeast cells transformed with *MaGRAS12* and *MaGRAS34* genes showed significant tolerance under 30% PEG–6000 treatments, especially under 100,000 times dilution, and the yeast cells transformed with the *MaGRAS33* gene showed drought and salt tolerance under 100,000 times dilution. We observed that the yeast cells transformed with *MaGRAS33* gene showed significant tolerance under 5 M NaCl treatment, especially under 1,000,000 times dilution; followed by the *MaGRAS12*, the *MaGRAS34* transformed yeast cells had no difference with the control. 

## 3. Discussion

*GRAS* genes widely exist in plants and play an important role in plant development and various physiological processes [30]. Genomic identification and the evolutionary relationship of GRAS family has been explored in many species with the rapid development of whole–genome sequencing technologies. Nevertheless, the identification of GRAS gene family and the study of gene function in *M. albus* has not been previously reported. Therefore, this study confirmed and comprehensively analyzed the members of GRAS family in *M. albus*, which allowed us to study the evolution of *M. albus* GRAS family and speculate the biological functions of some unknown *MaGRAS* genes. 

In this study, we identified and characterized 55 *MaGRAS* genes based on *M. albus* genome database, which were close to those of rice (60) [9] and alfalfa (68) [11], lower than those of soybean (117) [38] and wheat (153) [10] and higher than those of *A. thaliana* (34) [38] and barley (*Hordeum vulgare*) (34) [41]. This result was also paralleled to the genome size of different species, which shows that there is a positive correlation between the number of GRAS family genes and the genome size of the species. Evolutionary analysis showed that the MaGRAS proteins fit into eight subfamilies. Among the subfamilies, LISCL was the largest subfamily with 12 *MaGRAS* genes, and the PAT1 and DELLA subfamilies also consisted of multiple genes. MaGRAS proteins had been identified in every sub–population of *A. thaliana*, which indicates that these *GRAS* genes may play some fundamental biological functions during the long–term evolution of *M. albus* [42].

Studies have shown that gene duplication was the evolutionary force behind the expansion of the GRAS gene family [43]. In this study, all *MaGRAS* genes were unevenly distributed on eight chromosomes. Most of which are located on chromosome 4 (12 genes) and 7 (11 genes), and the number of *MaGRAS* genes on chromosome 6 is the least (2 genes). Interestingly, three groups of tandemly duplicated *MaGRAS* genes were found on chromosomes 4 and 7 (two/chr4, one/chr7). In addition to tandem duplications, a considerable number of *MaGRAS* family genes were derived from segmental duplication events, 10 pairs of duplicated segments in *MaGRAS* genes were evenly distributed on eight chromosomes. Gene fragment duplication can expand the number of *MaGRAS* genes in different subgroups, and many orthologous *MaGRAS* genes are produced on different chromosomes. The *MaGRAS* genes involved in segmental duplication events are mainly distributed in SHR (two genes), HAM (four genes), LISCL (two genes), PAT1 (eight genes) and SCR (three genes) subfamilies, among which *MaGRAS* genes from group PAT1 are distributed on different chromosomes (*MaGRAS2* and *MaGRAS*5 on chromosome 1, *MaGRAS6* and *MaGRAS9* on chromosome 2, *MaGRAS26* on chromosome 4, *MaGRAS38* on chromosome 5, *MaGRAS53* and *MaGRAS55* on chromosome 8) and are products of gene segmental duplication events. Studies have shown that gene duplication events are common in GRAS gene family. For example, *GRAS2* and *GRAS34* in *A. thaliana* [9], *GRAS15* and *GRAS53* in tomato [44] and *GRAS17* and *GRAS60* in rice [9] were identified as duplicated genes, which further confirmed that gene duplication may be a mechanism for the expansion of GRAS family members. 

In addition, the pattern analysis of the exon–intron of gene families can provide additional insights into their evolution [44]. It has been reported that the main reason for the absence of intron genes in gene families is the rapid duplication after horizontal gene transfer at the bacterial level [45]. Consistent with reports from other species, such as *A. thaliana* (67.6%) [9], tomato (77.4%) [45] and populus (54.7%) [42], the exon–intron structure analysis of the GRAS gene family in *M. albus* shows that most *MaGRAS* genes lack introns (87%) (Figure 4B). The high proportion of intronless genes in the gene family can further indicate the close evolutionary relationship of these family proteins [44]. Besides GRAS gene family, intronless genes have also been found in other gene families of plants, such as small auxin up–regulated RNAs (SAUR) [46] and F–box transcription factor [47]. Meanwhile, the change of introns in the process of evolution can be considered as a necessary way for gene families to acquire new gene functions [48]. Compared with most the genes of soybean PAT1 subfamily, which contain introns in 5′UTRS (untranslated region) [38], genes of PAT1 subfamily in *M. albus* do not contain introns. There are two genes in LISCL subfamily, including two introns and four introns, respectively, which indicates that the structure of *GRAS* genes is species–specific. Moreover, GRAS proteins belonging to the same taxonomic branch show a common evolutionary origin and have conserved motifs related to their functions [43]. These specific conserved motifs ensure the interaction of GRAS proteins and the characteristics of DNA binding modifications [49]. In this study, the 20 motifs predicted by MEME are classified into 5 conserved domains: LRHI, VHIID, LRHII, PFYRE and SAW are located at C–terminal of GRAS proteins (Figure 4C). Interestingly, MEME motifs in the five C–terminal conserved domains fluctuate with different subfamilies, especially the 2, 4, 12, 13 motifs are not present in every MaGRAS protein with SAW conserved domain, which may correspond to different functions of MaGRAS proteins in different subfamilies. Most MaGRAS proteins contain five conserved domains at the C–terminal, but the HAM subfamily (*Malbus0503484.1*) lacks the conserved domain of LRHII, the LISCL subfamily (*Malbus0503351.1*) lacks the conserved domain of VHIID, and the DELLA subfamily (*Malbus0704150.1*) lacks the conserved domains of LRHII, PFYRE and SAW. Generally speaking, the structural relationship between MaGRAS proteins can indicate their functional similarities and differences. Furthermore, we predicted the protein interactions of different subfamilies of GRAS in *M. albus*. The *MaGRAS14* gene of HAM subfamily can interact with multiple subfamilies, which may be the hub for regulating the functions of different proteins. *MaGRAS51* can interact with the nodulation–related transcription factor *MaGRAS29*. *MaGRAS51* may be involved in the formation and development of nodules in *M. albus*, but this needs further experimental verification.

*GRAS* genes are widely participated in regulating plant development and various stress responses [50,51]. In this study, the cis–acting elements in the 2000bp promoter regions upstream of *MaGRAS* genes were identified (Figure 5). It was found that the phytohormone response elements (auxin, gibberellin and methyl jasmonate, etc.) and abiotic stresses elements (defense, low temperature and drought, etc.) existed widely, and some elements related to plant growth and development (meristem expression, etc.) were also found. It is suggested that *GRAS* genes of *M. albus* play an important role in regulating plant development and responding to different stresses. Studies have shown that *GRAS* genes are expressed in many plant organs and tissues, including flowers, fruits, leaves, stems, roots and nodules [52], and the expression levels of *GRAS* genes will change according to plant development stage, species and environmental conditions [21]. In our study, tissue expression analysis showed that 14 *MaGRAS* genes had no discernible expression levels in 5 tissues (Supplementary Table S3), which indicated that these genes may have lost their functions in the process of evolution. A total of 27 (49.1%) *MaGRAS* genes were expressed in flowers, seeds, leaves, stems and roots tissues according to *M. albus* transcription analysis, indicating that these genes may participate in the regulation of plant growth and development. Noticeably, the gene expression of HAM, SHR, SCR and PAT1 subfamilies was the highest in all five tissues during the development of *M. albus* (Figure 6). *MaGRAS52* (orthologous to *AtPAT1*) and *MaGRAS38* (orthologous to *AtSCL13* and *AtSCL21*) in the PAT1 subfamily were highly transcribed in leaves, and they are functionally involved in the signal transduction of photosensitive pigments [53,54]. *AtSCL3*, an orthology of *MaGRAS48* (SCL3 subfamily), showed high expression in flowers, indicating that they might play a role in pollination/fertilization [55]. *MaGRAS12* (orthologous to *AtSHR*) in the SHR subfamily and *MaGRAS46* (orthologous to *AtSCR*) in the SCR subfamily were highly expressed in roots, which may be involved in the growth and development of roots [12,13]. According to the results of qRT–PCR analysis (Figure 6), *MaGRAS36* (orthologous to *At5g41920.1*, *At3g54220.1*) and *MaGRAS19* (orthologous to *At4g08250.1*) were specifically expressed in nodule, which may be involved in signal transduction in the process of nodulation. In summary, MaGRAS proteins are involved in different processes of plant development, which can lay a foundation for further research on the growth and development of *M. albus*.

At present, many studies have shown that *GRAS* genes have potential regulatory effects under various abiotic stresses [56]. In soybean (*Glycine max*), *GmGRAS37* was significantly upregulated under drought and salt stresses, and the overexpression of *GmGRAS37* gene improved soybean resistance [57]. *BrLA*S gene in *Brassica rapa* can limit water loss by controlling stomatal opening, thus playing a role in drought resistance [58]. In our study, most of the genes can be induced obviously in roots but not in leaves (Figure 7), which indicates that the expression of *M. albus GRAS* genes are tissue specific. Additionally, we found that 52.7% and 40.0% *MaGRAS* genes were expressed in roots and stems under ABA and abiotic stresses, respectively, and most of *MaGRAS* genes belong to PAT1, LISCL and DELLA subfamilies. Notably, nine *MaGRAS* genes were up–regulated under ABA and abiotic stresses, and four of them (*MaGRAS2*, *MaGRAS23*, *MaGRAS38* and *MaGRAS55*) were distributed in PAT1 subfamily. The expression levels of PAT1 subfamily genes *Gh_D01G0564* and *Gh_A04G0196* in cotton (*Gossypium hirsutum*) increased obviously under salt, drought, cold and high temperature stress, which indicated that PAT1 subfamily genes played an important regulatory role in plant response to abiotic stresses [51]. Meanwhile, compared to control samples, there were 46 (83.6%) *MaGRAS* genes differentially expressed in the roots and shoots under under ABA, drought and salt stress treatments, and these *MaGRAS* genes were evenly distributed in 8 subfamilies. In addition, the expression of *TaTLP2–B* and *TaCRK68–A* can significantly enhance the tolerance of yeast cells under abiotic stress conditions, and the yeast heterologous expression is an important method to verify the function of genes under abiotic stress conditions [59,60]. In this study, *MaGRAS12* and *MaGRAS34* can significantly enhanced the tolerance of yeast cells under drought treatment, while *MaGRAS33* can significantly enhance the tolerance of yeast cells under salt treatment (Figure 8). In future studies, these genes can be used to develop transgenic plants tolerant to abiotic stresses. These results indicated that *MaGRAS* genes play vital roles in the regulation of *M. albus* response to ABA and abiotic stresses.

## 4. Materials and Methods

### 4.1. Identification of the GRAS Genes in M. albus

The genome sequences [36] and RNA–seq data [61,62] of *M. albus* was obtained from College of Pastoral Agriculture Science and Technology, Lanzhou University. GRAS gene sequences of *A. thaliana* and *M. truncatula* are downloaded from the Phytozome database (https://phytozome.jgi.doe.gov/pz/portal.html, accessed on 5 February 2022) for searching and identifying GRAS sequences in *M. albus* genome. The putative GRAS genes were submitted to CDD (https://www.ncbi.nlm.nih.gov/Structure/bwrpsb/bwrpsb.cgi, accessed on 5 February 2022), Pfam (http://pfam.xfam.org/, accessed on 5 February 2022) and SMART (http://smart.embl-heidelberg.de/, accessed on 5 February 2022); all GRAS proteins were identified by the GRAS domain (PF03514) in the Hidden Markov Model (HMM) profile. The online tool EXPASY (http://web.expasy.org/protparam/, accessed on 5 February 2022) [63] was used to analyze the molecular weights, amino acid residues, grand average of hydropathicity and isoelectric points were of *MaGRAS* genes. The online tool CELLO v2.5 system (http://cello.life.nctu.edu.tw, accessed on 5 February 2022) was used to predict the subcellular localization of *MaGRAS* genes [64].

### 4.2. Phylogenetic Analysis and Classification of MaGRASs

The full–length amino acid sequences of GRASs derived from 55 *MaGRASs*, 34 *AtGRASs* and 68 *MtGRASs* were used for phylogenetic analysis. Sequence alignment was performed using Ctlustal X with the default settings [65]. The neighbor–joining phylogenetic tree was constructed by using MEGA 7.0 software, and 1000 boot tests were performed to support statistical reliability [66]. According to orthology with *AtGRASs* and *MtGRASs*, the *MaGRAS* genes were further divided into 8 subgroups.

### 4.3. Chromosomal Mapping and Gene Duplication Analysis of MaGRAS Genes

The location information of all *MaGRAS* genes in the *M. albus* genome was extracted and submitted to the online tool MAP Gene 2 Chromosome V2 (http://mg2c.iask.in/mg2c_v2.1/, accessed on 10 February 2022) to display the *MaGRAS* genes on the corresponding chromosomal locations. The collinearity of 55 *MaGRAS* genes was analyzed by TBtools software [67].

### 4.4. Analysis of Gene Structures and Motif Composition of MaGRAS Genes

The full–length amino acid sequences of 55 *MaGRASs* were used for construct unrooted phylogenetic tree, and the genes were further divided into 8 different subfamilies. The MEME was employed (http://meme-suite.org/, accessed on 15 February 2022) to identify the conserved motifs of MaGRAS proteins in *M. albus* by set MEME to find 20 motifs. [68]. TBtools was used to analyze and visualize the exon–intron structure of MaGRAS members in *M. albus* genome [67].

### 4.5. The Prediction of Protein–Protein for MaGRAS Proteins

The prediction of protein–protein interactions of MaGRAS protein subfamilies was performed by the online tool STRING 11.5 (https://cn.string-db.org/cgi/input?sessionId=P9OZVCkjeo8u&input_page_show_search=on, accessed on 27 June 2022), with reference to the protein–protein interactions of the GRAS proteins of *M. truncatula*.

### 4.6. Identification of Putative Cis–Elements in MaGRAS Genes

Sequences of 2000bp from the upstream promoters of the *MaGRAS* genes were extracted by TBtools software [67], and submitted to PlantCARE website (http://bioinformatics.psb.ugent.be/webtools/plantcare/html/, accessed on 21 February 2022) to identify the cis–acting elements in the promoter regions [69]. 

### 4.7. Transcriptome Analysis for Tissue–Specific Expression and Stress Treatment

Transcriptome data of the *MaGRAS* genes in different tissues (roots, leaves, seeds, flowers and stems) and under ABA, drought and salt stress in *M. albus* were obtained from our previous study [61,62]. The analysis of RNA–seq data were the same as those in a previous study [62]. The *M. albus* genes expression levels were estimated based on fragments per kilobase of exon model per million mapped reads (FPKM) [61]. TBtools software was used to show a heat map and a Venn diagram of the *MaGRAS* genes expression profile [67].

### 4.8. Quantitative Real–Time PCR Analysis for Tissue–Specific Expression and Stress Treatment

Using TransZol reagent (TransGen Biotech, Beijing, China), the total RNA was extracted from stress treatment of the shoots (leaves and stems) and roots of control and treated seedlings (ABA, drought and salt stress) and from different tissues of the roots, nodules, stems, flowers and leaves samples during the flowering stage. Reverse transcription and cDNA synthesis were performed using the cDNA synthesis kit (Sangon Biotech Ltd., Shanghai, China) from 1 μg of total RNA. The quantitative primers designed by PerlPrimer software (Supplementary Table S5). The qRT–PCR experiment was carried out with Hieff^®^ qPCR SYBR^®^ Green Master Mix (No Rox) (Yeasen Biotech Co., Ltd., Shanghai, China) on a CFX96 Real–Time PCR Detection System (Bio–Rad, Los Angeles, CA, USA). The qRT–PCR reaction procedure is 40 cycles of 95 °C for 5 s, 60 °C for 15 s, and 72 °C for 34 s. The expression level of *Maβ–tubulin* gene was used as control. The relative expression level of each gene was calculated by 2^−ΔΔCT^ method [70], and the experiment was set up for 3 biological repetitions.

### 4.9. Functional Verification of Heterologous Expression Genes in Yeast

The full–length coding sequences of *MaGRAS12*, *MaGRAS33* and *MaGRAS34* were amplified using the cDNA as the template, and ClonExpress^®^ MultiS One Step Cloning Kit (Vazyme Biotech Co., Ltd., Nanjing, China) was used to connect the pYES2 expression vector. Specific amplification primers used in this experiment are listed in Supplementary Table S5. The empty pYES2 plasmid and recombinant pYES2–*MaGRAS12*, *MaGRAS33* and *MaGRAS34* plasmids were transferred into *Saccharomyces cerevisiae* strain *INVSc1*. The method is consistent with previous studies [62,71]. The 4 yeast cultures were independently grown in synthetic complete (SC)–Ura liquid medium containing 2% (*m*/*v*) galactose at 30 °C for 36 h up to A600 = 0.4. Then, yeast was collected and adjusted with SC–Ura containing 2% galactose and cultured to A600 = 1 for stress analysis. The same number of yeast cells were resuspended in 30% PEG–6000 and 5 M NaCl. Then, the serial dilutions (100, 10^−1^, 10^−2,^ 10^−3^, 10^−4^, 10^−5^, 10^−6^) were spotted on SC–Ura agar plates and incubated at 30 °C for three days. As a control, yeast A600 = 1 was also spotted on SC–Ura agar plate with the same dilution as the treatment and grew at 30 °C for three days. The colony growth was observed, and the expression of binding protein was recorded by taking photos.

## 5. Conclusions

This study represents the first comprehensive identification and analysis of the GRAS gene family of *M. albus*. All 55 *MaGRAS* genes are divided into 8 subfamilies and unevenly distributed on chromosomes 1 to 8 in *M. albus*. GRAS proteins of the same subfamily have similar protein motifs. Gene structure analysis showed that *MaGRAS* genes had few introns and were highly conserved in *M. albus*. The promoter region contains many plant hormone response elements and stress response elements. In addition, gene expression analysis using RNA–seq data and qRT–PCR showed that *MaGRAS* genes played an important role in plant growth and development, ABA and abiotic stress responses. The remarkable tolerance of yeast cells expressing *MaGRAS12*, *MaGRAS33* and *MaGRAS34* further established their roles in drought and salt stresses. Taken together, these results provide useful insights for future research on the function of *MaGRAS* genes in stress response and the development of transgenic plants.

## Data Availability

The genomic data of *M. ablus* are openly available in NCBI (NCBI BioProject ID PRJNA674670).

## Supplementary Materials

The following supporting information can be downloaded at: https://www.mdpi.com/article/10.3390/ijms23137403/s1.
