# Supplementary material for: Genome–Wide Identification of the GRAS Family Genes in Melilotus albus and Expression Analysis under Various Tissues and Abiotic Stresses
Source: Int J Mol Sci. 2022 Jul 3;23(13):7403. doi: 10.3390/ijms23137403 (PMC9267034; doi:10.3390/ijms23137403)
Supplement: Supplementary file 1 [file ijms-23-07403-s001.zip › ijms-1787251-Supplementary Material.pdf]

Genome-wide identification of the GRAS family genes in *Melilotus albus* and expression analysis under various tissues and abiotic stresses

Shengsheng Wang, Zhen Duan, Qi Yan, Fan Wu, Pei Zhou and Jiyu Zhang*

State Key Laboratory of Grassland Agro-ecosystems, Key Laboratory of Grassland Livestock Industry Innovation, Ministry of Agriculture and Rural Affairs; College of Pastoral Agriculture Science and Technology, Lanzhou University, Lanzhou 730020, China

Shengsheng Wang: shshwang21@lzu.edu.cn

Zhen Duan: duanzh12@lzu.edu.cn

Qi Yan: yanq16@lzu.edu.cn

Fan Wu: wuf15@lzu.edu.cn

Pei Zhou: zhoup2017@lzu.edu.cn

Jiyu Zhang: zhangjy@lzu.edu.cn

* Corresponding author: Jiyu Zhang (zhangjy@lzu.edu.cn)

Supplementary Materials

Figures:

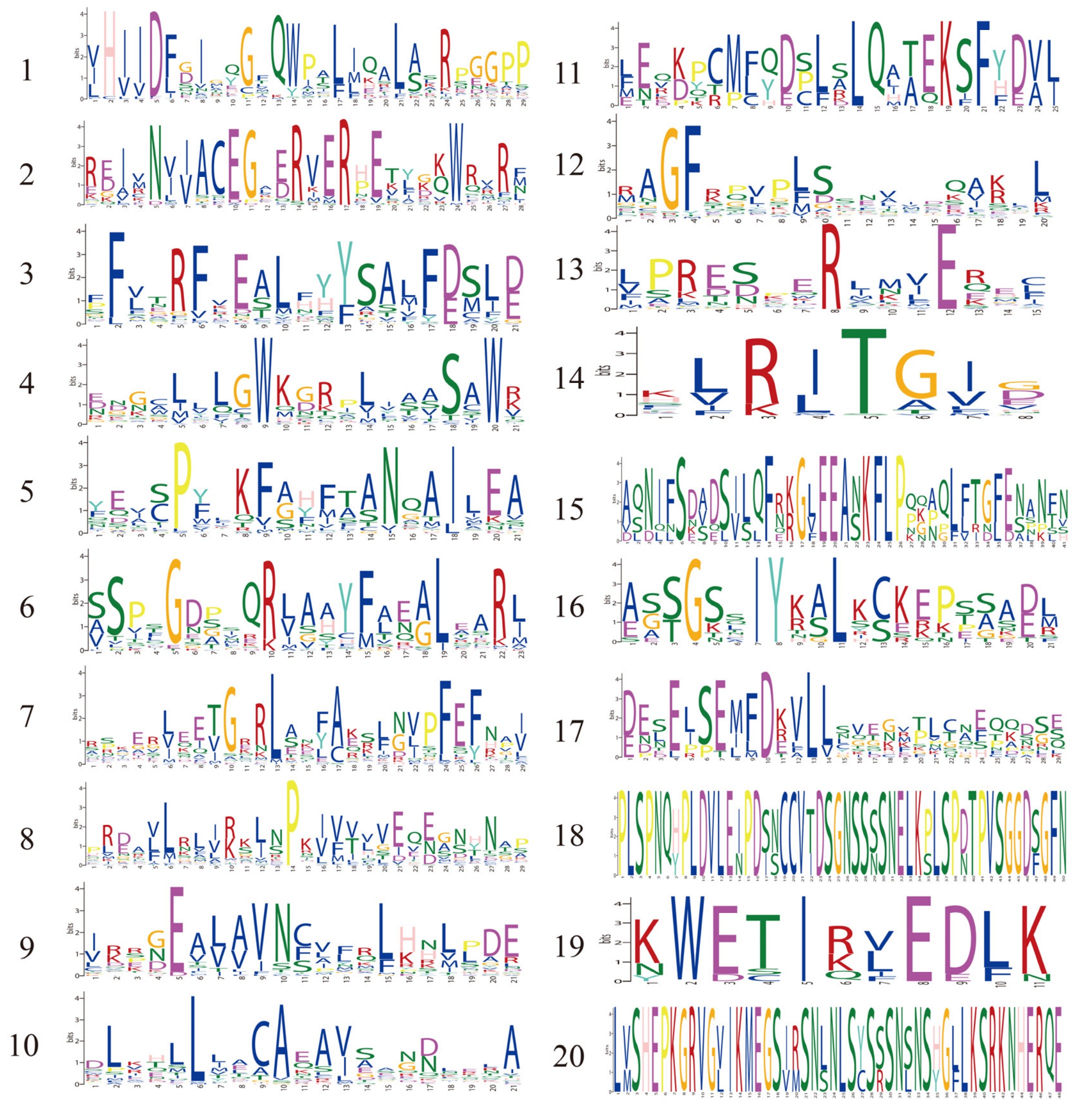

Supplementary Figure S1. 20 web logo representing the motif of *MaGRASs*. The logo consists of stacks of symbols, one stack for each position in the sequence. The overall height of the stack indicates the sequence conservation at that position, while the height of symbols within the stack indicates the relative frequency of each amino acid at that position.

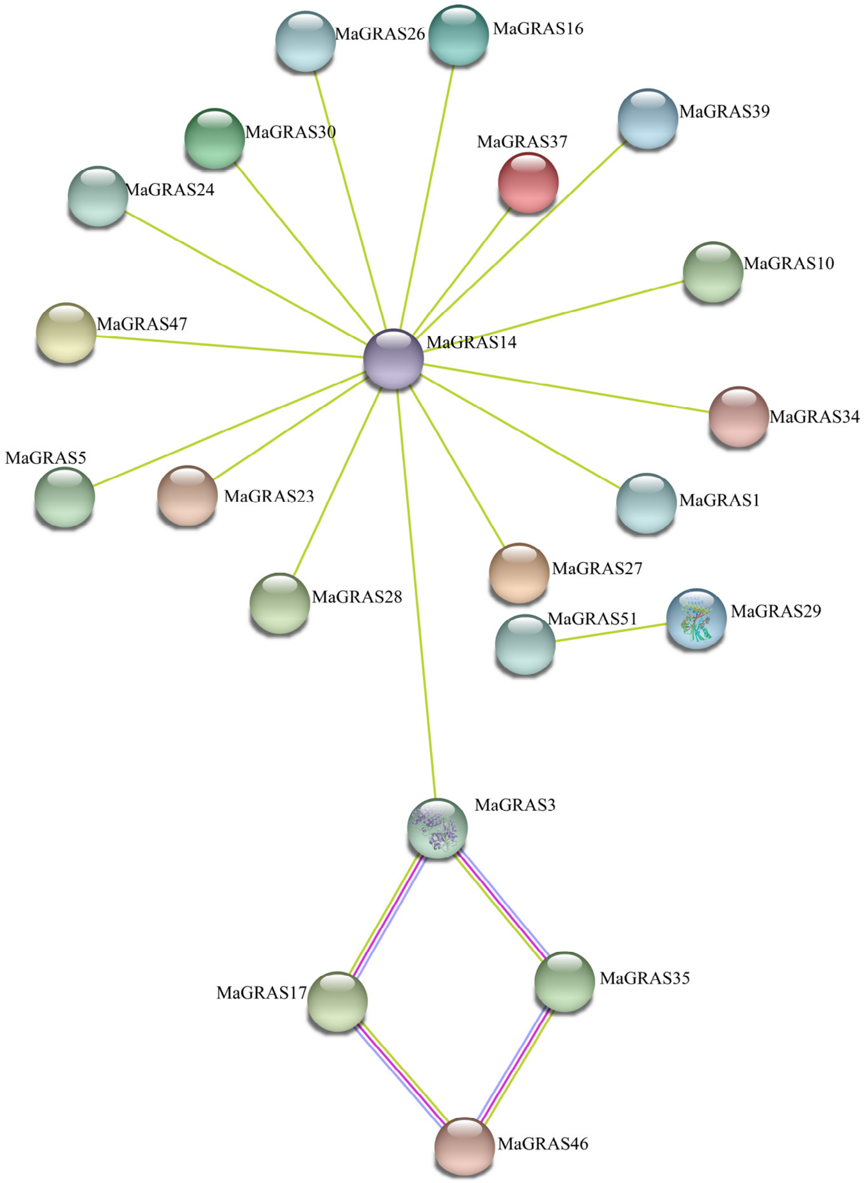

Supplementary Figure S2. Prediction of protein-protein interactions of the GRAS subfamily of *M. albus*.

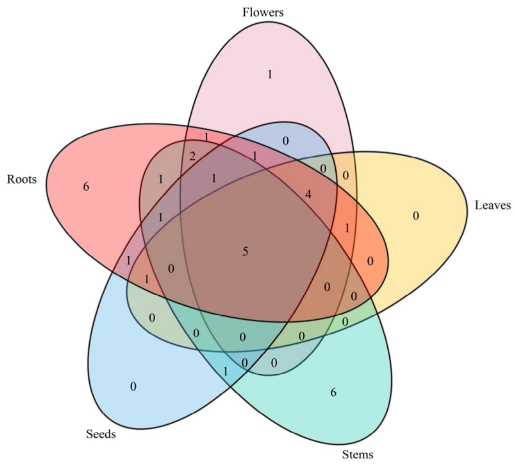

Supplementary Figure S3. Venn diagram showed overlap of highly expressed *MaGRAS* genes (FPKM ≥10) in different tissues.

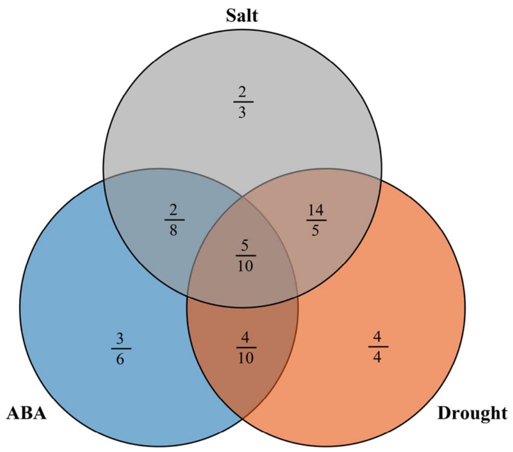

Supplementary Figure S4. Venn diagram showing the overlap of differentially expressed *MaGRAS* genes under ABA and abiotic stress, and the letters on the line and below the line indicate the differentially expressed *MaGRAS* gene numbers in the shoots and roots, respectively.

Tables:

Supplementary Table S1. Characteristics of *GRAS* genes in *M. Albus*.

Gene name	Gene ID	Chromosome location	Number of amino acids	Molecular weight	Isoelectric points	Grand average of hydropathicity	Length of CDS	Subcellular localization	Subgroup
MaGRAS17	Malbus0400444.1	Chr4:3855862-3857217	451	51953.75	5.6	-0.486	1356	nucl	SHR
MaGRAS24	Malbus0401252.1	Chr4:18168844-18170220	458	51636.2	5.58	-0.119	1377	cyto	SHR
MaGRAS34	Malbus0503672.1	Chr5:104092698-104094110	470	52827.74	5.82	-0.191	1413	cyto	SHR
MaGRAS40	Malbus0601405.1	Chr6:25257693-25259171	492	55948.53	5.48	-0.452	1479	chlo	SHR
MaGRAS35	Malbus0503690.1	Chr5:104636438-104637955	505	57829.27	5.01	-0.526	1518	nucl	SHR
MaGRAS29	Malbus0500746.1	Chr5:12379956-12381632	558	62247.91	5.62	-0.693	1677	nucl	SHR
MaGRAS4	Malbus0102889.1	Chr1:41321418-41322782	454	51945.09	5.69	-0.349	1365	nucl	LAS
MaGRAS48	Malbus0702939.1	Chr7:92452694-92454343	549	60997.36	4.96	-0.297	1650	nucl	LAS
MaGRAS50	Malbus0704150.1	Chr7:108311964-108312598	155	17065.2	5.67	-0.244	468	nucl	DELLA
MaGRAS25	Malbus0402383.1	Chr4:60279050-60280552	500	55808.17	5.12	-0.177	1503	cyto	DELLA
MaGRAS37	Malbus0504010.1	Chr5:109822072-109823649	525	59182.76	6.24	-0.17	1578	cyto	DELLA
MaGRAS49	Malbus0704097.1	Chr7:107540357-107541976	539	59697.24	5.45	-0.287	1620	nucl	DELLA
MaGRAS30	Malbus0502166.1	Chr5:52391697-52393325	537	60877.87	4.84	-0.257	1629	cyto	DELLA
MaGRAS8	Malbus0203178.1	Chr2:45745951-45747747	598	65672.63	5.02	-0.325	1797	cyto	DELLA
MaGRAS11	Malbus0300868.1	Chr3:34137498-34140646	675	74957.12	5.63	-0.386	2028	nucl	DELLA
MaGRAS51	Malbus0704714.1	Chr7:115353500-115355023	507	58190.35	4.82	-0.116	1524	nucl	HAM
MaGRAS7	Malbus0202609.1	Chr2:33375219-33376745	508	56374.94	5.53	-0.35	1527	cyto	HAM
MaGRAS54	Malbus0802657.1	Chr8:63818692-63820230	512	57165.13	4.91	-0.262	1539	cyto	HAM
MaGRAS33	Malbus0503484.1	Chr5:100215619-100217232	537	59950.92	5.57	-0.236	1614	chlo	HAM
MaGRAS14	Malbus0302319.1	Chr3:92874622-92876343	573	65399.67	4.82	-0.351	1722	nucl	HAM
MaGRAS41	Malbus0700855.1	Chr7:23293300-23295450	716	80461.53	5.61	-0.349	2151	nucl	HAM
MaGRAS12	Malbus0301355.1	Chr3:58962509-58964974	737	82063.56	5.35	-0.295	2214	nucl	HAM

MaGRAS32	Malbus0503351.1	Chr5:97284330-97285892	451	50777.27	5.53	-0.447	1470	nucl	LISCL
MaGRAS21	Malbus0400537.1	Chr4:5185313-5187076	587	67531.26	5.45	-0.38	1764	nucl	LISCL
MaGRAS31	Malbus0503131.1	Chr5:91621798-91623701	589	67350.25	7.57	-0.213	1770	chlo	LISCL
MaGRAS44	Malbus0702293.1	Chr7:80880234-80882051	605	69550.67	5.75	-0.53	1818	nucl	LISCL
MaGRAS22	Malbus0400538.1	Chr4:5190338-5192203	722	82155.18	5.53	-0.286	1866	nucl	LISCL
MaGRAS18	Malbus0400534.1	Chr4:5139267-5141228	653	73798.33	5.59	-0.479	1962	nucl	LISCL
MaGRAS19	Malbus0400535.1	Chr4:5142441-5144411	656	74493.85	5.32	-0.517	1971	nucl	LISCL
MaGRAS45	Malbus0702294.1	Chr7:80893807-80895792	661	74205.46	6.06	-0.495	1986	nucl	LISCL
MaGRAS43	Malbus0702292.1	Chr7:80875756-80877924	722	82155.18	5.53	-0.587	2169	nucl	LISCL
MaGRAS42	Malbus0702291.1	Chr7:80872127-80874331	734	83258.63	6.32	-0.556	2205	nucl	LISCL
MaGRAS20	Malbus0400536.1	Chr4:5151174-5153399	741	83832.38	5.49	-0.485	2226	nucl	LISCL
MaGRAS13	Malbus0301980.1	Chr3:81640926-81643154	742	83834.03	6.24	-0.425	2229	nucl	LISCL
MaGRAS53	Malbus0800311.1	Chr8:4384940-4386538	532	59874.7	5.17	-0.312	1599	nucl	PAT1
MaGRAS26	Malbus0403118.1	Chr4:92439035-92440651	538	60657.33	5.95	-0.365	1617	chlo	PAT1
MaGRAS9	Malbus0203583.1	Chr2:56151461-56153089	542	61672.33	5.19	-0.42	1629	nucl	PAT1
MaGRAS52	Malbus0800099.1	Chr8:1759997-1761631	544	61084.07	5.81	-0.323	1635	chlo	PAT1
MaGRAS38	Malbus0505561.1	Chr5:130254156-130255796	546	61366.22	5.74	-0.371	1641	nucl	PAT1
MaGRAS6	Malbus0201875.1	Chr2:22663788-22665485	565	63918.87	5.2	-0.453	1698	chlo	PAT1
MaGRAS23	Malbus0400986.1	Chr4:13227530-13229272	544	61084.07	5.81	-0.357	1743	nucl	PAT1
MaGRAS5	Malbus0105102.1	Chr1:122667590-122669365	563	62732.21	4.81	-0.436	1776	nucl	PAT1
MaGRAS55	Malbus0803098.1	Chr8:84306050-84308014	654	72719.59	6.63	-0.49	1965	chlo	PAT1
MaGRAS2	Malbus0102084.1	Chr1:27315431-27317602	723	80309.83	5.66	-0.481	2172	chlo	PAT1
MaGRAS10	Malbus0204960.1	Chr2:114833358-114836458	438	49829.63	6.8	-0.147	1317	cyto	SCL3
MaGRAS47	Malbus0702874.1	Chr7:91397554-91398978	474	53661.85	6.48	-0.177	1425	nucl	SCL3
MaGRAS39	Malbus0600285.1	Chr6:3913171-3914619	482	54139.06	5.51	-0.19	1449	nucl	SCL3
MaGRAS46	Malbus0702863.1	Chr7:91218957-91220300	447	49358.85	5.38	-0.228	1344	mito	SCR

MaGRAS3	Malbus0102533.1	Chr1:35041926-35043377	483	54790.3	5.67	-0.204	1452	nucl	SCR
MaGRAS1	Malbus0101661.1	Chr1:21715313-21716785	490	56251	6.37	-0.191	1473	nucl	SCR
MaGRAS16	Malbus0304697.1	Chr3:126809531-126811156	531	61574.71	5.66	-0.37	1626	nucl	SCR
MaGRAS27	Malbus0403782.1	Chr4:110897157-110898941	594	68025.38	5.06	-0.295	1785	nucl	SCR
MaGRAS28	Malbus0403783.1	Chr4:110903425-110905215	596	68654.03	5.25	-0.35	1791	nucl	SCR
MaGRAS36	Malbus0503963.1	Chr5:109194048-109196081	677	75221.95	5.51	-0.455	2034	chlo	SCR
MaGRAS15	Malbus0302550.1	Chr3:97687685-97690716	819	90545.57	6.08	-0.508	2460	nucl	SCR

Supplementary Table S2. Analyses the motifs in *M. albus* GRAS proteins from the MEME website [1].

Motif	E-value	Sites	Width	Best possible match	GRAS-specific C-terminal domains
1	9.4e-744	53	29	VHIIDFDIGQGFQWPALIQALASRPGGPP	VHIID
2	1.9e-571	39	28	REIVNVIACEGAERVERHETYGKWRVRF	SAW
3	7.8e-421	51	21	FFVTRFVEALFYYSALFDSLD	PFYRE
4	2.0e-390	52	21	ENGCLLLGWKGRPLIAASAWR	SAW
5	5.1e-385	51	21	YEVSPYLKFAHFTANQAILEA	
6	2.9e-367	45	23	SSPSGDPIQRLAAYFAEALEARJ	LRHI
7	1.0e-371	48	29	RSKERLZETGRRLAKFAKSLNVPFEFNAI	LRHII
8	2.6e-369	49	29	PRDAVLRLIRKJNPKIVVVVEQEGNYNAP	PFYRE
9	1.9e-246	43	21	IRRGEALAVNCVFRLHNLLDE	LRHII
10	7.9e-238	55	21	DLKHLLJACAZAVSANBLELA	LRHI
11	4.2e-096	11	25	LEQKPCMFQDPLSLQATEKSFYDVJ	
12	1.6e-112	48	20	RAGFRPVPLSSNVIDQAKLL	SAW
13	1.8e-094	36	15	LPRESKERJMVEREC	SAW
14	1.6e-085	45	8	KLRITGID	VHIID
15	5.2e-069	7	41	AQNIFSDADSILQFRKGLEEANKFLPQKAQLFTGFENANFN	
16	3.0e-055	12	21	ASSGSSIYKALKCKEPSSADL	
17	1.3e-043	12	29	DESELSEMFDKVLLSVEGKPLCAEQQDSE	
18	1.7e-036	3	50	PLSPNQHPLDVLEIPDSNCCVTDSGNSSSSNELKPLSPDTPVSGGDSGFN	
19	3.2e-035	10	11	KWETIRLEDLK	
20	3.6e-032	3	48	LVSHEPKGRVGVIKMEGSIRSNLNLSYSSSNSNSHGLLKSRKNHERQE	

Supplementary Table S3. Expression pattern of *MaGRAS* genes in different organ.

Gene ID	Gene Name	Leaf	Root	Stem	Flower	Seed
Malbus0401252.1	MaGRAS7	0.400045	0.420973	0.26499	0.53909	0.124199
Malbus0400444.1	MaGRAS54	0.3203	0.198943	0.270387	0.615131	1.489007
Malbus0503672.1	MaGRAS14	0.359841	1.704517	0.668214	0.55334	0.686715
Malbus0503690.1	MaGRAS51	0	0.29981	0	0	0.47552
Malbus0601405.1	MaGRAS12	15.89499	23.76835	15.10414	19.16728	24.57583
Malbus0500746.1	MaGRAS41	4.298491	12.15113	9.546952	11.15561	12.47423
Malbus0102533.1	MaGRAS33	16.84492	10.64287	15.90398	34.35559	10.50252
Malbus0702863.1	MaGRAS3	0	0.271247	0.4233	0.662765	0
Malbus0302550.1	MaGRAS46	5.057094	16.25217	7.29307	8.813203	4.653436
Malbus0503963.1	MaGRAS15	0.692034	2.541432	8.458577	7.719423	18.87458
Malbus0101661.1	MaGRAS36	0.667517	17.91621	12.61276	14.32783	0.771307
Malbus0403783.1	MaGRAS1	0.50905	0	0.536327	0.18134	1.187262
Malbus0403782.1	MaGRAS28	0.435133	0.456557	0.126429	0.341376	0.346927
Malbus0304697.1	MaGRAS27	0.45708	0.504197	0.474073	0.450854	0.453623
Malbus0702874.1	MaGRAS16	0.41335	0.459875	0	0.210691	0.264122
Malbus0600285.1	MaGRAS4	0.53658	0	0.364465	0.332496	0.254662
Malbus0204960.1	MaGRAS48	6.594983	13.92625	19.18301	12.40702	7.567597
Malbus0105102.1	MaGRAS5	0	69.44597	28.37868	14.56354	18.53842
Malbus0403118.1	MaGRAS26	2.532255	2.354607	1.502069	1.442957	2.009266
Malbus0400986.1	MaGRAS23	13.50318	24.93132	28.44593	23.19816	38.95246
Malbus0203583.1	MaGRAS9	16.49112	35.42916	14.04345	13.48918	9.333943
Malbus0201875.1	MaGRAS6	0.316005	14.91192	1.480411	0.375189	0.653531

Malbus0102084.1	MaGRAS2	7.725554	33.55504	6.645661	24.82644	6.946609
Malbus0505561.1	MaGRAS38	28.73511	138.112	15.46331	13.20476	8.275305
Malbus0800311.1	MaGRAS53	1.665492	15.74168	2.731053	5.396427	11.41095
Malbus0800099.1	MaGRAS52	13.50318	24.93132	28.44593	23.19816	38.95246
Malbus0803098.1	MaGRAS55	7.331471	33.10082	11.27496	41.87194	15.47519
Malbus0702291.1	MaGRAS42	7.002843	2.7943	4.325431	8.208419	1.736526
Malbus0301980.1	MaGRAS13	7.702959	4.283106	4.196538	4.154538	3.247467
Malbus0400536.1	MaGRAS20	6.229766	12.72078	3.636158	8.982319	5.651591
Malbus0503131.1	MaGRAS31	6.229766	12.72078	3.636158	8.982319	5.651591
Malbus0503351.1	MaGRAS32	0.18562	0	0	0	0
Malbus0702293.1	MaGRAS44	1.443656	5.629392	2.657879	1.624184	1.637462
Malbus0702292.1	MaGRAS43	1.90545	17.95996	2.678234	2.354711	3.411629
Malbus0400537.1	MaGRAS21	1.347795	14.52392	0.759947	0.788066	0.375339
Malbus0400538.1	MaGRAS22	5.197869	1.203206	3.585441	2.610841	2.50015
Malbus0400535.1	MaGRAS19	5.065737	28.34783	9.315928	2.586494	8.158397
Malbus0400534.1	MaGRAS18	0.344237	11.21758	0.630424	0.200533	2.154042
Malbus0702294.1	MaGRAS45	9.401725	11.49191	8.626331	8.600211	16.70747
Malbus0102889.1	MaGRAS24	0.66269	0	0.19294	0.46499	0.369448
Malbus0702939.1	MaGRAS17	0.368059	5.354429	12.31819	2.314483	0
Malbus0202609.1	MaGRAS34	0.46613	0.179052	0	0.7688	0.488572
Malbus0802657.1	MaGRAS35	0.551271	0.456136	5.394822	3.054147	3.372018
Malbus0302319.1	MaGRAS40	5.946141	8.398582	7.487167	7.541841	6.128639
Malbus0704714.1	MaGRAS29	0.48179	0.389197	0.31476	0.490497	0.241296
Malbus0301355.1	MaGRAS8	31.23272	22.18742	35.53674	67.69514	13.25611
Malbus0700855.1	MaGRAS49	44.67056	48.34482	56.65321	37.00652	22.75587
Malbus0503484.1	MaGRAS50	0.297169	1.419682	1.086555	1.028771	0
Malbus0203178.1	MaGRAS25	2.141788	1.411137	1.460064	12.02941	0.738533
Malbus0704097.1	MaGRAS37	0.13665	0	0.22342	0.297174	0
Malbus0704150.1	MaGRAS11	0.34116	0.346607	0.635463	0.425363	0
Malbus0402383.1	MaGRAS30	0.45731	0.988337	0	0.29032	0.290042
Malbus0504010.1	MaGRAS47	2.909387	5.43214	3.078301	2.48972	1.142406
Malbus0300868.1	MaGRAS39	2.494206	9.187843	3.885858	4.457513	6.798117
Malbus0502166.1	MaGRAS10	0.545517	0.898775	0.667995	0.730774	1.86652

Supplementary Table S4. The transcriptome data of *MaGRAS* genes in shoot and root under abiotic stress.

Gene ID	Gene Name	Drought						Salt						ABA					
		Root			Shoot			Root			Shoot			Root			Shoot		
		CK	3h	24h	CK	3h	24h	CK	3h	24h	CK	3h	24h	CK	1h	24h	CK	1h	24h
Malbus0704097.1	MaGRAS49	104.97	57.23	68.30	82.99	71.68	95.88	109.62	78.50	57.57	89.31	65.46	61.54	109.62	132.19	128.58	89.31	125.72	119.58
Malbus0105102.1	MaGRAS5	35.95	118.76	52.10	11.42	19.26	30.84	177.48	201.51	232.45	31.54	35.00	40.72	177.48	128.01	156.96	31.54	30.87	34.18
Malbus0800099.1	MaGRAS52	28.26	48.66	52.10	14.59	30.32	39.55	68.43	35.17	26.25	9.97	7.65	5.21	68.43	66.20	93.92	9.97	18.39	19.72
Malbus0102084.1	MaGRAS2	38.05	30.32	41.85	16.14	18.18	20.98	11.26	17.35	16.05	4.23	3.10	6.78	11.26	39.34	36.83	4.23	12.07	18.38
Malbus0301355.1	MaGRAS12	42.40	22.93	40.03	29.86	26.25	20.31	9.86	14.02	11.46	10.88	4.74	10.23	9.86	34.56	13.02	10.88	11.56	21.40
Malbus0803098.1	MaGRAS55	27.34	37.56	39.89	12.62	12.13	20.01	26.96	22.47	28.17	6.55	5.82	8.91	26.96	44.92	45.11	6.55	14.36	17.08
Malbus0203583.1	MaGRAS9	28.03	61.70	38.02	22.55	11.38	18.54	26.77	36.10	42.48	11.28	6.23	12.40	26.77	26.13	20.15	11.28	10.45	18.96
Malbus0400535.1	MaGRAS19	30.97	138.81	36.21	3.04	7.28	7.87	39.76	83.46	38.69	6.80	8.14	11.94	39.76	90.67	28.10	6.80	12.02	14.16
Malbus0503484.1	MaGRAS33	33.74	19.41	33.41	20.31	12.45	20.28	10.69	16.86	12.55	4.64	3.11	4.29	10.69	29.45	26.83	4.64	10.33	14.76
Malbus0203178.1	MaGRAS8	42.37	33.35	31.99	71.22	60.22	41.18	14.34	16.12	20.55	20.51	10.20	8.66	14.34	37.98	16.75	20.51	36.77	40.28
Malbus0400986.1	MaGRAS23	7.63	84.76	28.29	7.43	24.06	69.52	71.07	67.62	198.51	28.99	34.01	47.58	71.07	23.55	65.98	28.99	44.45	40.12
Malbus0601405.1	MaGRAS40	26.23	16.51	24.40	9.90	7.61	3.72	5.61	5.97	2.11	1.15	0.62	0.38	5.61	30.94	13.94	1.15	3.07	3.11
Malbus0505561.1	MaGRAS38	14.74	81.05	19.89	7.05	12.96	16.77	107.02	154.80	130.87	12.60	18.64	15.17	107.02	127.01	50.40	12.60	16.08	11.78
Malbus0400536.1	MaGRAS20	15.04	15.49	17.75	10.38	8.41	11.28	22.06	23.52	17.93	7.95	5.70	10.27	22.06	24.84	11.05	7.95	9.76	11.95
Malbus0700855.1	MaGRAS41	15.84	13.48	15.48	10.19	9.11	10.29	4.62	7.95	5.43	4.98	2.90	5.05	4.62	13.06	6.32	4.98	6.25	8.73
Malbus0702294.1	MaGRAS45	13.32	25.54	15.32	9.53	8.82	15.72	49.29	41.27	45.02	14.76	18.59	27.16	49.29	41.83	42.98	14.76	13.06	16.82
Malbus0503963.1	MaGRAS36	23.11	9.78	14.50	12.34	16.56	4.17	0.39	2.05	0.34	0.09	0.45	0.11	0.39	15.40	0.85	0.09	5.44	0.28
Malbus0403118.1	MaGRAS26	3.68	8.15	14.24	3.70	1.06	1.67	5.13	4.71	13.03	1.60	0.90	1.25	5.13	1.74	5.78	1.60	1.38	2.31
Malbus0201875.1	MaGRAS6	17.00	6.37	12.98	0.73	0.65	0.58	39.24	16.19	11.17	0.48	0.23	0.13	39.24	26.29	39.59	0.48	0.46	0.59
Malbus0800311.1	MaGRAS53	8.12	33.15	12.83	3.97	4.24	7.91	23.87	29.08	26.66	1.40	2.78	2.95	23.87	9.41	14.25	1.40	1.80	1.28
Malbus0702939.1	MaGRAS48	14.10	7.43	12.42	24.27	8.96	9.46	1.97	2.15	1.73	5.54	3.85	2.85	1.97	8.51	3.48	5.54	13.53	25.79

Malbus0702292.1	MaGRAS43	10.02	35.83	11.36	1.29	0.87	2.55	27.44	36.55	19.45	4.07	2.81	3.42	27.44	41.15	14.03	4.07	1.89	3.16
Malbus0600285.1	MaGRAS39	10.25	4.66	10.14	6.75	7.39	14.41	10.00	6.10	3.97	16.92	12.52	21.05	10.00	9.67	9.52	16.92	6.04	21.13
Malbus0702863.1	MaGRAS46	9.94	7.74	8.79	8.01	11.11	34.62	7.66	7.48	14.10	9.53	11.95	15.31	7.66	8.75	13.94	9.53	11.91	22.94
Malbus0702874.1	MaGRAS47	4.99	3.40	8.47	3.88	3.32	2.56	1.81	1.53	1.00	1.23	0.98	0.97	1.81	0.82	0.96	1.23	1.36	1.96
Malbus0302550.1	MaGRAS15	9.64	9.56	7.84	11.63	8.15	4.77	4.35	5.39	3.01	2.60	0.77	0.60	4.35	18.08	4.91	2.60	3.93	3.73
Malbus0400534.1	MaGRAS18	8.59	25.32	7.11	0.08	0.32	0.35	32.99	28.07	16.90	0.77	0.69	0.24	32.99	40.16	21.28	0.77	0.41	0.41
Malbus0400444.1	MaGRAS17	5.06	3.70	4.43	4.25	2.73	2.47	2.49	2.73	2.04	1.03	1.11	0.66	2.49	1.39	1.38	1.03	1.86	1.44
Malbus0402383.1	MaGRAS25	6.46	2.03	4.20	5.04	3.90	1.33	0.14	0.24	0.07	0.33	0.16	0.16	0.14	3.40	0.29	0.33	0.84	1.09
Malbus0702293.1	MaGRAS44	4.75	3.32	4.01	1.52	2.12	1.71	7.74	5.58	4.21	3.64	2.72	4.31	7.74	6.98	7.26	3.64	2.60	4.60
Malbus0503690.1	MaGRAS35	4.80	6.88	3.76	3.58	3.55	2.29	1.14	1.48	0.57	0.49	0.52	0.19	1.14	11.76	2.93	0.49	1.27	0.82
Malbus0301980.1	MaGRAS13	2.26	4.17	3.68	4.26	8.33	5.46	6.34	8.43	6.90	5.94	3.73	6.42	6.34	4.76	5.13	5.94	5.17	7.09
Malbus0802657.1	MaGRAS54	21.96	4.73	2.43	0.19	0.12	0.08	0.12	0.05	0.03	0.01	0.01	0.03	0.12	8.83	0.19	0.01	0.18	0.11
Malbus0702291.1	MaGRAS42	2.37	2.79	2.17	3.59	5.78	2.07	0.55	0.80	0.46	1.17	1.26	0.63	0.55	1.77	0.55	1.17	3.57	3.43
Malbus0503672.1	MaGRAS34	1.83	5.25	2.14	0.75	0.83	0.00	2.80	3.16	2.59	0.02	0.01	0.00	2.80	5.95	4.56	0.02	0.00	0.01
Malbus0704150.1	MaGRAS50	0.99	0.90	1.23	0.51	0.76	0.40	0.61	0.69	0.65	0.29	0.17	0.16	0.61	0.70	0.85	0.29	0.48	0.45
Malbus0401252.1	MaGRAS24	1.32	0.25	1.22	4.63	1.44	0.19	0.06	0.18	0.07	1.07	0.06	0.21	0.06	1.71	0.14	1.07	0.47	1.60
Malbus0500746.1	MaGRAS29	1.72	0.98	1.04	0.00	0.00	0.00	3.49	2.15	1.00	0.00	0.00	0.00	3.49	3.23	3.43	0.00	0.00	0.00
Malbus0400537.1	MaGRAS21	1.61	16.01	1.00	1.34	1.27	0.68	9.17	16.88	19.40	1.29	1.51	1.86	9.17	2.57	0.62	1.29	0.13	0.51
Malbus0204960.1	MaGRAS10	0.66	0.52	0.80	0.56	0.84	0.55	1.68	1.61	1.35	0.64	0.38	0.67	1.68	0.64	1.02	0.64	0.50	0.93
Malbus0704714.1	MaGRAS51	3.07	0.82	0.70	0.01	0.02	0.04	0.12	0.55	0.11	0.02	0.02	0.01	0.12	3.06	0.11	0.02	0.00	0.00
Malbus0102533.1	MaGRAS3	1.10	0.74	0.66	0.00	0.00	0.02	0.06	0.19	0.04	0.01	0.00	0.00	0.06	0.25	0.02	0.01	0.01	0.00
Malbus0403783.1	MaGRAS28	1.91	0.96	0.58	0.32	0.07	0.10	9.36	8.82	9.75	0.12	0.07	0.09	9.36	3.92	4.78	0.12	0.12	0.08
Malbus0502166.1	MaGRAS30	0.64	1.24	0.52	0.00	0.01	0.00	0.23	0.51	0.84	0.00	0.00	0.01	0.23	0.09	0.17	0.00	0.00	0.00
Malbus0403782.1	MaGRAS27	1.28	1.65	0.43	0.06	0.11	0.17	2.01	3.72	3.31	0.26	0.24	0.36	2.01	1.65	0.83	0.26	0.11	0.10
Malbus0400538.1	MaGRAS22	0.34	1.35	0.29	17.49	10.78	10.83	5.30	8.12	4.50	1.10	1.03	5.97	5.30	5.51	5.57	1.10	4.75	16.00
Malbus0300868.1	MaGRAS11	0.09	0.03	0.28	0.02	0.01	0.01	0.00	0.02	0.00	0.00	0.00	0.00	0.00	0.00	0.00	0.00	0.01	0.00

Malbus0304697.1	MaGRAS16	0.41	0.24	0.26	0.02	0.05	0.02	3.22	3.35	2.40	0.43	0.25	0.19	3.22	3.31	2.13	0.43	0.26	0.51
Malbus0202609.1	MaGRAS7	0.84	0.53	0.25	0.12	0.01	0.03	0.90	0.40	0.24	0.04	0.01	0.04	0.90	0.40	0.76	0.04	0.12	0.08
Malbus0302319.1	MaGRAS14	1.11	0.62	0.22	0.07	0.08	0.82	1.54	7.41	2.62	0.11	0.32	1.35	1.54	3.69	1.61	0.11	0.12	0.17
Malbus0102889.1	MaGRAS4	0.24	0.05	0.05	1.34	1.19	0.97	0.07	0.04	0.04	0.23	0.21	0.06	0.07	0.35	0.07	0.23	0.50	0.27
Malbus0101661.1	MaGRAS1	0.00	0.01	0.00	0.02	0.02	0.00	0.00	0.01	0.00	0.01	0.00	0.00	0.00	0.00	0.00	0.01	0.00	0.00
Malbus0503131.1	MaGRAS31	0.00	0.00	0.00	0.00	0.00	0.00	0.00	0.00	0.00	0.00	0.00	0.00	0.00	0.00	0.00	0.00	0.00	0.00
Malbus0503351.1	MaGRAS32	0.00	0.00	0.00	0.00	0.00	0.00	0.00	0.00	0.01	0.00	0.00	0.00	0.00	0.01	0.00	0.00	0.00	0.00
Malbus0504010.1	MaGRAS37	0.01	0.04	0.00	0.00	0.02	0.00	0.01	0.00	0.01	0.01	0.00	0.01	0.01	0.00	0.00	0.01	0.01	0.00

Supplementary Table S5. Primers used in this study.

Primers	Sequences
Maβ-tubulin-F	CCTTGGTGGTGGAACTGGT
Maβ-tubulin-R	GGAGATGGGAACACTGAGAAAG
MaGRAS7-F	AACGACACACTTGCTGCTTT
MaGRAS7-R	GGGCCACGGATTCGATTATG
MaGRAS8-F	TCGAGCTCTAACTCGAACCC
MaGRAS8-R	CCTCCACTGACCCAAAGTCT
MaGRAS12-F	GAGTTGTGACCGAACCGATG
MaGRAS12-R	AGCTTCTCAATTGCCGGTTG
MaGRAS17-F	GCTGGTTGGTCACTTGTTGT
MaGRAS17-R	AGGGTTTCCATGTTGATGCC
MaGRAS19-F	AGATGACTTGGAGCGCAAAC
MaGRAS19-R	GCAGCTGTTGCTATTGCTCT
MaGRAS29-F	AAGCTTTGACAAACCAGCGT
MaGRAS29-R	CGCCATCAATTGCATCCTCT
MaGRAS33-F	ACCACCACTCAGAACATCGT
MaGRAS33-R	GTAGCACAAGGTGGTGGTTG
MaGRAS34-F	GCGCTTCGAGGTTTAGATCC
MaGRAS34-R	CGGCTTCATACCATTGCCTT
MaGRAS36-F	ACTAGGCTTTGGCCTCATGT
MaGRAS36-R	AGCCCTCAACAGCATCTCAT
MaGRAS38-F	CAAATCGCACAGGGAACAC
MaGRAS38-R	CAAACGGAACTCCACGAGAC
MaGRAS47-F	GCTGCTGAACCTTCTCAGTG
MaGRAS47-R	GCTACCTGGTCCAGAACCTC
MaGRAS48-F	AACGACGCATGCCCATATTC
MaGRAS48-R	AATGCAGCCCATTGAATCCC
MaGRAS12-pYES2-F	taccgagctcggatcATGAATGAGATGAAGGCTATGC

MaGRAS12-pYES2-R	gatgcggccctctagTCAGCATCTCCAAGTCGAAAC
MaGRAS33-pET32a-F	taccgagctcggatcATGAGAGTTCCTCCTCCTG
MaGRAS33-pET32a-R	gatgcggccctctagCTAACACCTCCAAGCTGAC
MaGRAS34-pET32a-F	taccgagctcggatcATGATGCAATTCACACATTCAC
MaGRAS34-pET32a-R	gatgcggccctctagTTAAGCAGGCAACCAAGC
